# Motivational dynamics of German Salafist jihadists: A multi-methodical in-depth study of three paradigmatic cases

**DOI:** 10.3389/fpsyg.2022.1009222

**Published:** 2022-11-15

**Authors:** Mika Josephine Moeller, Phil C. Langer, Herbert Scheithauer

**Affiliations:** ^1^Department of Education and Psychology, Freie Universität Berlin, Berlin, Germany; ^2^Department of Social Psychology, International Psychoanalytic University, Berlin, Germany; ^3^Zentrum Technik und Gesellschaft, Technische Universität Berlin, Berlin, Germany; ^4^Center for Transdisciplinary Gender Studies, Humboldt-University Berlin, Berlin, Germany

**Keywords:** qualitative case study, Salafist jihadism, radicalization, motivation, cognitive dissonance, in-depth hermeneutic

## Abstract

Individuals belonging to terrorist organizations accept and often use violence as an instrument of their strategies to achieve their goals. The present study focuses on the motivational dynamics of three contrastively selected paradigmatic cases of extremists that grew up in Germany, joined and supported terrorist organizations abroad, and later disengaged and distanced themselves from the jihadist ideology. An innovative multi-methodical approach was applied to the interviews that combines a biographical reconstruction of the lived experiences with a psychoanalytically informed interpretation of the narratives. First, the biographical trajectories were analyzed on the manifest level: How have the former terrorists experienced their own pathways? What were relevant factors for their engagement in and disengagement from terrorism? Second, to gain a deeper understanding of the unconscious motivational dynamics for involvement in terrorism, key sequences of the narrative interviews were interpreted scenically in a psychoanalytical interpretation group: How did the interviewees express their lived experiences (and why in this particular way)? What latent meanings can be extrapolated that provide deep insights into the motivational backgrounds of their decisions? Based on the results of the triangulation process, characterizing structural hypotheses about case dynamics including protective and risk factors are presented and implications for prevention and intervention approaches are given.

## Introduction

In the civil war raging in Syria and Iraq since 2011, foreign terrorists played a crucial role in supporting jihadist terrorist groups (Dawson, 2021). With a total of 1,150 Germans who left the war and crisis zone in Syria and Iraq, Germany plays a relevant role concerning support for and involvement in terrorist groups abroad. Around 260 German foreign fighters have lost their lives, and only one-third has since returned (Bundesamt für Verfassungsschutz, 2021, 2022, p. 202). At least 100 of them were verifiable actively involved in combat or attended combat training (Bundesamt für Verfassungsschutz, 2021, p. 202). Until 2018, at least 61 cases brought to court by the Chief Federal Prosecutor at the Federal Court of Justice (in German *Generalbundesanwalt*) resulted in convictions for membership of or support for a (foreign) terrorist group and/or planning to commit serious acts of violent subversion (Deutscher Bundestag, 2018; Pelzer and Moeller, 2020 p. 3).

Joining or supporting a terrorist group is often preceded by a process of radicalization, which can be understood as a multi-layered and complex psychosocial process that is influenced by a variety of factors and mechanisms (Horgan, 2009). The motives for joining terrorist organizations are as heterogeneous as the motives to leave those. Research on how and why western foreign fighters left to the war zone in Syria/Iraq still lacks consistent and adequate empirical data (Dawson, 2021). Quantitative studies found frequencies of rather general, distal characteristics of Western foreign fighters, such as a large part are male, in their middle until the late 20s when departure to the warzone (Bundeskriminalamt et al., 2016), they do not differ psychopathologically from the general population (see, e.g., Silke, 2003; Victoroff, 2005; Gill and Corner, 2017), often have limited prospects (Coolsaet, 2016; Roy, 2017) as well as delinquent experiences (Dawson, 2021). However, the phenomenon of radicalization to terrorist engagement is highly diverse. Although studies have found sociodemographic characteristics to be significant in some cases, Wolfowicz et al. (2020, p. 436) rate their overall impact as relatively small and also note that some of the results are heterogeneous over different studies, which may be due to changing characteristics of terrorist offenders or simply reflect the wide diversity of these individuals (p. 409). In conclusion, there is consensus that no typical terrorist profile exists (see, e.g., Silke, 2003; Horgan, 2008; Gill et al., 2014; Klausen et al., 2020).

The Institute for Economics and Peace (2022 p 6) defines terrorism broadly as “the systematic threat or use of violence, by non-state actors, whether for or in opposition to established authority, with the intention of communicating a political, religious or ideological message to a group larger than the victim group, by generating fear and so altering (or attempting to alter) the behavior of the larger group.” Thus, radicalization toward joining or supporting a terrorist group does not necessarily lead to one’s own use of violence but at least to acceptance of it as a legitimate pattern of action. However, according to the Global Terrorism Index 2022, only four Islamist terrorist organizations were responsible for the majority of deaths of all terrorist groups worldwide in 2021: the so-called Islamic State (IS), Al-Shabaab, the Taliban, and Jamaat Nusrat Al-Islam wal Muslimeen (JNIM) (Institute for Economics and Peace, 2022, p. 15).

For research purposes, individuals who departed to a foreign country to join a terrorist group are a “hard-to-reach group” as most stay in the foreign country or die in combat. Especially for qualitative research, a fundamental problem, therefore, lies in a lack of access to empirical primary data and evidence-based research, which often remains anecdotal (see, e.g., Leimbach, 2020), as well as in a “lack of analytical rigor and transparency” (Lynch and Joyce, 2018; Morrison, 2020, p. 7). Little is known about the motivational and intrapsychic processes involved in radicalization and the decision to join and fight for a foreign terrorist group. First-hand interviews are essential to gain an understanding of the motivations of terrorist actors (see, e.g., Morrison, 2020). Studies on members of terrorist groups usually rather refer to practical core motives (see, e.g., Bouzar, 2017) or personal goals to which a person may be committed (see, e.g., Kruglanski et al., 2014) than to motivations. As there is no typical profile of a terrorist, we argue that there is also no typical motivation for people who join (and leave) terrorist groups. Moreover, many more individuals with the same personal or sociodemographic characteristics do not join terrorist groups than those who do. Instead, a more holistic case approach should be conducted: What drives individuals in their lives, how do they see their agencies in decision making, and what can we learn about individuals’ radicalization and disengagement from terrorism on that base? To approach this question, a psychoanalytically oriented approach is particularly suitable, as it enables us to relate an individual’s conscious and unconscious intrapsychic dynamics to biographical pathways and thus to contribute to a deeper understanding of the phenomenon of (de)radicalization processes.

### The present study

The present study aims to analyze the lived experiences of three homegrown German former Salafist extremists who joined terrorist groups abroad and to identify the complex motivational dynamics along their biographical pathways of (de)radicalization based on a multi-methodical qualitative analysis of their narrations. This is a new perspective for understanding and researching processes that lead individuals to both engage in and disengage from terrorism.

The three cases we have studied traveled to a war and crisis zone and were later convicted for joining and/or supporting terrorist groups abroad. Some of them completed combat training. According to the current state of knowledge, they were not involved in active combat missions. However, there was at least a commitment to do so. They are thus exemplary for a large part of jihadists or, more specifically, Jihad returnees. In contrast to those killed in the combat zone or those who remain there, these individuals are to be actively reintegrated into society in the coming years. Therefore, this analysis provides important information on the motivational dynamics underlying radicalization and the willingness to use (mass) violence and to turn away from such aspirations.

This article is structured as follows: The data collection process is described and the mixed-methodological approach that combines thematic analysis with a psychoanalytically informed interpretation is outlined in Section “Materials and methods.” In Section “Results,” the key themes that emerged in the analytic process are presented and the complex motivational dynamics are set out in detail by using in-depth case reconstructions. Finally, the findings are placed in a sociopsychological tradition of cognitive dissonance theory and implications for both prevention and intervention in the context of jihadist (de)radicalization are provided in the concluding Section “Discussion and Conclusion.”

## Materials and methods

### Data collection

This study is based on selected data collected within the national research project “PrADera—Practice-oriented analysis of deradicalization processes” by Emser et al. (2022), which was approved and funded within the framework of the National Prevention Program against Islamist Extremism and aimed to gain a better understanding of distancing from Salafist extremism. The underlying data protection concept for the study with human participants was reviewed and approved by the data protection office of the Technische Universität Berlin and complies with the data protection requirements of the EU General Data Protection Regulation (GDPR). The participants provided their written informed consent to participate in the study. Narrative biographical interviews (see, e.g., Schütze, 1976; Rosenthal, 1995), with former extremists of the Salafist scene, were conducted in 2020. Interview candidates were acquired directly, or contacts were established *via* so-called gatekeepers (lawyers, preachers, exit counselors) or personal recommendations (snowball system). Cooperating with the institutional data protection officer, a comprehensive data protection concept was designed that complies with the data protection requirements of the GDPR, to which this study is also committed. Due to the sensitivity of the topic and the collected data in terms of Article 9 of the GDPR, a comprehensive risk analysis was also carried out as part of the data protection concept. Participation in the interviews was voluntary and was conducted under strict data protection criteria regarding the documentation, storage, and anonymization of the data, to ensure the anonymity of the interview candidates. The interview and recording started only after the interview candidates took note of all relevant information, measures to ensure data protection, their personal rights, and their informed consent. According to the GDPR and general research ethics, they were informed that they have the right to revoke their consent. Only after the consent form was signed, the interview process began. The consent-revocation form remained with the interview candidates.

The narrative interview is an open and non-structured interview procedure. It begins with an explicit request to talk about one’s experiences. In the opening question, the interviewer defines the subject of the expected narrative (Kleemann et al., 2013). The general opening question was: “You were recently released from prison [and also participated in an exit program] and a lot has changed in your life in the last few years. We would like to understand what has changed in your life and how. You can just start wherever it feels right for you.” Following the narrative interview rules, the interview candidates were asked to talk freely whereby the interviewer took a neutral role and did not intervene by asking questions or comments. Questions of understanding were only asked afterward (see, e.g., Kleemann et al., 2013).

### Analytical approach

The selection criteria for the in-depth analysis were as follows: The first selection criterion was that the interview candidates were born in Germany or at least came to Germany before the age of six (primary socialization in Germany). This threshold was set to ensure that all interview candidates attended elementary school in Germany, as school can be assumed to be an important socialization and living environment. The second selection criterion was departure to a war and crisis zone and a related conviction for supporting/joining a terrorist organization abroad [§129a “Forming terrorist organizations” and §129b “Foreign criminal and terrorist organizations; confiscation” of the German Criminal Code (in German *Strafgesetzbuch*)]. On this basis, three cases were selected as suitable for further in-depth analysis for the present study.

One person each was born in Germany (*Daniel)*, born in another country (*Omar)*, and born in Germany with a second-generation migration background (*Arjan)*, but all grew up in Germany since early childhood. All three cases supported or joined jihadist organizations abroad and have traveled to war and crisis zones, for which they were later convicted in Germany. At the time of the interviews, all interviewees had already been released from prison and perceived themselves as distanced from Salafist jihadist ideology. The interviews were conducted face to face in public places without large public traffic (e.g., university facilities). The duration of the interviews was between 1.5 and 2.5 h (Daniel: 2.5 h, Omar: 1.5 h, Arjan: 2 h).

To reconstruct the actual life course of individuals, on the one hand, and to analyze intrapsychic dynamics of the individuals, on the other hand, a multi-stage methodological procedure that combines a biographical reconstruction of the lived experiences with a psychoanalytically informed interpretation of the narratives was developed and applied to the interview transcripts.

#### Reconstruction of biographical pathways

First, according to the biographical–analytical approach of Rosenthal (1995), all accessible objective biographical dates per case were collected chronologically in a timeline to reconstruct the former jihadist’s biographical pathways through (de)radicalization. This included, for example, birth dates, all accessible information about the educational and professional career, as well as information about relationships, family, significant events, religious activism, or repression. All objective biographical data were thematically clustered by the first author into comparable categories: Following or disrupting institutional norms (e.g., attending vs. quitting vocational training), following or disrupting social norms (e.g., marriage, or the birth of own children vs. divorce), Delinquency, Repression in the sense of repressive measures taken by the police in accordance with the German Code of Criminal Procedure (e.g., Detention or passport revocation), (re)adaption of Islam, Salafist activism (e.g., engagement in a Salafist group, Quran distribution), jihadist activism (e.g., joining or supporting a foreign jihadist terrorist group), and disengagement of jihadist activism (e.g., leaving a jihadist terrorist group or distancing from jihadist ideology). In this way, the complexity of the different biographical trajectories was reduced, and the anonymity of the interviewees was guaranteed both for the further analytical process and for the subsequent presentation of cases and results.

The biographical timeline was additionally clustered into characterizing phases to visualize the biographical pathway of (de)radicalization: before (re)adaption of Islam, (re)adaption of Islam, Salafist activism, jihadist activism, disengagement from activism, and reintegration into society. These phases are to be understood as artificial and analytical divisions, as we assume that the (de)radicalization process is fluid and that phases may overlap and do not necessarily have a start and end point.

To be able to compare and reflect on the time periods within and between cases, a time bar was created that indicates the time intervals between different biographical events in years.

#### Reconstruction of the lived experiences

Oriented to the approach of reflexive thematic analysis by Braun and Clarke (2006, 2019), which is well established in psychological research, the interview transcripts of the three former terrorists were open coded with the software program MAXQDA. As the first author was involved in the interview processes and had previously evaluated them in a research project on reasons for leaving Salafist extremism (Emser et al., 2022), the author was already very familiar with the interviews. All narratives concerning one’s own path were open and multi-coded on a manifest as well as a latent level by the first author.

The first research question was: How do the former jihadists see their own agency in different stages of their biography and how have they perceived their experiences? Thus, first it was analyzed in a theory-based way how the individuals represented the progression of their life course and which active or reactive attitude they took toward their biography. Schütze (1976) distinguishes, among other process structures, between self-initiated patterns of action (in German *biografische Handlungsmuster*) and passive curves of progression (in German *Verlaufskurven*). Based on that, it was coded whether the interviewee described a decision that led to a biographical experience as actively or passively made [e.g., “I didn’t want to go to school anymore and dropped” (active) vs. “I was then expelled from school” (passive)].

To gain a deeper understanding of intrapsychic dynamics, all narratives of biographical experiences were then open coded in a data-driven way. Therefore, all narrations containing evaluations of biographical experiences, perceptions, needs, desires, or conflicts that emerged in the narrations were additionally open coded. These sequences were coded exhaustively, meaning that as many codes were assigned to a sequence as resulted from the material. Later, the codes were summarized into emerging main and subthemes (Braun and Clarke, 2006). This process included re-coding of the interviews whenever a new theme was created that was previously not considered in the analysis or if a theme was eliminated. As suggested by Braun and Clarke (2006, p. 22, p. 22), the themes that emerged from the data were defined in relation to the research question, which sought to identify the lived experiences in the own life course. The constructed main themes are further described in results section: Sense of Belonging, Quest for Belonging, Quest for Significance, Empowerment, Excitement, Anxiety, and Pleasure. Subthemes were created for each of these main themes. The subthemes represent whether an intrapsychic perception that was coded with one of the main themes was expressed as either increasing or decreasing (or not relevant) for each biographical experience (see section “Sense of belonging”).

The coded main and subthemes and the information about the decision-making process were transformed into the chronological biographical timelines of the three cases. Therefore, for each case and for each biographical date, it was recorded whether and which intrapsychic phenomenon occurred in the narration (main theme), as well as a subcode of how it was perceived. According to the in-depth hermeneutic approach, this process was conducted intersubjectively by the first author (see, e.g., Haubl and Lohl, 2020). Based on the interpretations and codings of the first author, the process was discussed and reflected with a second researcher familiar with the in-depth hermeneutic approach and intersubjective qualitative research methods. In case of inconsistencies between the researchers, the main themes and subthemes were revised, i.e., differentiated, merged, or newly created until consistency was attained. This was done to ensure reliability in the sense of consistency of the coding and the resulting themes.

#### Psychoanalytically informed interpretation of the lived experiences

To further develop a more in-depth understanding of unconscious motivational dynamics for engagement in terrorism, key sequences of the narrative interviews were interpreted scenically (in German *szenisches Verstehen*; see, e.g., Lorenzer, 1970, 2013; König et al., 2018; Haubl and Lohl, 2020) in a psychoanalytical working interpretation group of five psychoanalytically trained researchers that were familiar with the methodology of in-depth hermeneutics. The interpretation group serves as a resonance space for decoding the latent level of meaning in relation to the manifest level of meaning (Abd-Al-Majeed et al., 2021, p. 27). As suggested by Hollway and Volmerg (2010), the sequences to be interpreted were selected by the first author based on an initial analysis of the entire transcript. The entire interview transcripts were only known by the first author. This was on the one hand due to data protection reasons arising from the data protection concept and on the other hand to achieve the highest possible objectivity and avoidance of bias due to background or contextual knowledge within the interpretation group. The sequences were completely anonymized, meaning that all specific information was not only pseudonymized but neutrally replaced [e.g., (foreign country); (association), (name of another person)] or removed from the sequence (e.g., […]). Sequences in which an association with the person might still have been possible were not selected for the analysis.

The selected sequences were openly discussed by the interpretation group. At first, the group exchanged first impressions. As invented by Lorenzer (1970), this unstructured process is led by *free associations* and *irritations* that occur to the interpreters, while they examined the interview transcripts. In these *key scenes*, the latent meaning that lies behind the manifest meaning can be revealed (Haubl and Lohl, 2020, p. 568). Scenic interpretation requires an attitude of *balanced attention*, as well as a reflection of emotions, affects, irritations, and free association of the interpreters (Haubl and Lohl, 2020; Abd-Al-Majeed et al., 2021, p. 26) and in other words abductive instead of deductive inference (König, 2018, p. 35; Reichertz, 1993). Thus, the in-depth hermeneutic process is based on the reflection of the intersubjectivity of the researchers (Haubl and Lohl, 2020). The objective of the analytical focus is not only on the research material, but also on the dynamic processes that take place in the interpretation group, which need to be critically reflected to ensure transparency and comprehensibility (Haubl and Lohl, 2020, p. 565). It was interpreted as follows: How did the interviewees express their lived experiences (and why in this particular way)? What latent meanings can be extrapolated that provide deep insights into the motivational backgrounds of their decisions? Following the group interpretation, conclusions can finally be drawn as to which contexts of meaning have a manifest and which have a latent character and how they relate to each other (König, 2018, p. 34).

#### Triangulation process

The results of the multi-level approach: (1) the objective biographical reconstruction of the life trajectories, (2) the reconstruction of the lived experiences, and (3) the psychoanalytic interpretation of the lived experiences, were triangulated to holistically reconstruct the three cases. On that basis, a holistic intrapsychic pathway model through radicalization and disengagement from jihadist activism was created for each case that enables to draw characterizing structural hypotheses per case about the individual pathways, personal agencies, as well as conscious and unconscious intrapsychic and motivational dynamics. The presented case reconstructions can be understood in terms of the second (*theoretical comprehension of the case reconstruction*) and the third field (*reflected writing of the case reconstruction*) of the in-depth hermeneutic understanding process (see, e.g., König, 2018, p. 34). The final case reconstructions are presented as *research vignettes*, which include a psychoanalytically informed interpretation of the findings including a critical reflection (see, e.g., Langer, 2016).

## Results

**FIGURE 1:**
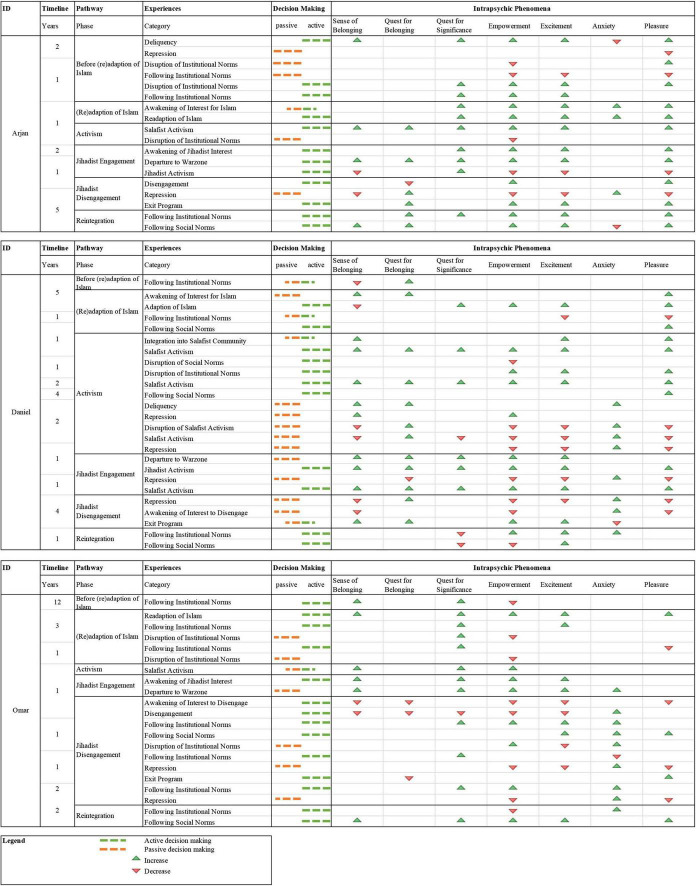
Multi-methodical case reconstruction of three paradigmatic cases of Salafist jihadist (de)radicalization. Reconstructions are visualized for an overall timeline, pathways of (de)radicalization, clustered biographical experiences, the responsibility for the decision making that led to it, and the relevance of intrapsychic phenomena during those experiences. *N* = 3; three male convicted homegrown German former jihadists.

To reconstruct the motivational dynamics over the life course of individuals who participated in and turned away from terrorism, a multi-methodical in-depth analysis was conducted. First, key themes reflecting intrapsychic phenomena that have been revealed from the interviews through the thematic analysis are presented. Reconstructions of the characteristic dynamics of intrapsychic phenomena and personal agencies through the biographical pathways are given per case. This is followed by triangulated concluding research vignettes, including individual case reconstructions of lived experiences, as well as results and reflections of the psychoanalytically induced interpretation. This dynamic approach is illustrated in Figure 1.

### Key themes

#### Sense of belonging

The theme *Sense of Belonging* reflects the actual perception at that time. In the interviews, the feeling of being part or belonging to a defined group, a family system, or a wider community was expressed in different ways. The interviewees described their integration into groups or communities (+) or how they felt as being a part of those (+): “*I then got to know other people in the scene, because you small talk, the world is small and people then came to me, ‘oh lion, good brother’ and stuff, I was further invited to various rallies* […]” (Arjan, pos. 82). In a broader way, the Sense of Belonging was also expressed more comprehensively: “*we are a community, we have to stick together, because the other countries are all watching, nobody helps*” (Omar, pos. 44), as well as described quite precisely: “*I find it interesting that everything (.), that my girlfriend, my wife now, was just the right one*” (Omar, pos. 601).

The feeling of being disconnected from a group or community (-) was often linked to conflicts: “*then I started going to Friday prayers, which I still do regularly and even there from some people you hear stupid comments, but I don’t pay attention to that, meanwhile there is nothing anymore*” (Omar, pos. 456) or “*there were many disputes [.] where then also of course (.) people change their behavior and then suddenly show their true faces* [.]” (Daniel, pos. 4).

#### Quest for belonging

In contrast to the above theme, the *Quest for Belonging* consists of a seeking or desire to belong to a group, community, or family system (+): “*What I was looking for in the end was people who., I just needed a role model, a big brother, brotherhood, loyalty, that’s what I needed*” (Arjan, pos. 73) or “*out of the prison cell for a bit. Getting to know other faces, other people*” (Arjan, pos. 261). The active Quest for Belonging and being in contact with others contrasts with an expressed attitude of not depending on community (-): “*Nobody talks much about Jihad, they were afraid themselves [.]. And then I informed myself*” (Arjan, pos. 90).

Another aspect of a decreasing seeking for belonging is the desire to distance oneself from a group or community (-): “*so then I was back in Germany (.) I also stayed away from the mosques for the time being, everybody was like “ey where were you (*…*), tell me, how was it,” I didn’t feel like talking to anyone about it* (.)” (Omar, pos. 212).

#### Quest for significance

As the Quest for Significance is already a well-established concept in radicalization research, meaning a “fundamental desire to matter, to be someone, to have respect” (see, e.g., Kruglanski et al., 2014, p. 73), its relevance also emerged in different ways in the interviews. It was represented as a longing for reputation and respect (+): “*Then, of course, I was bragging that I had been in* [a war and crisis zone]” (Arjan, pos. 206), a general seeking for being successful (+): “*Then, slowly I understood that I have to finish school*” (Arjan, pos. 42) or longing for purpose (+): “*no matter what, I have to help the Ummah, Ummah is the Islamic community, no matter how, the main thing is that I have to be on the spot and give my assistance*” (Omar, pos. 44). A decreasing Quest for Significance (-) was, for example, reflected by an expressed rejection of secular aspects: “*then we wanted to renounce us completely from the world*” (Daniel, pos. 69).

#### Empowerment

A feeling of Empowerment represents, in distinction to the Quest for Significance, an existing feeling of achieved significance or success (+): “*And then I felt immortal. And then I leaned against everyone who came against me*” (Arjan, pos. 30). The feeling of not being empowered (-) or not having the capacities to achieve something (-) is reflected in the feeling of decreasing Empowerment: “*then I didn’t manage to graduate. (.), then I was also expelled from school*” (Arjan, pos. 21). Another expression of not feeling empowered is the perception of having no option for action (-): “*every day the same routine, you just sit there, (*…*) and nothing happens*” (Omar, pos. 146).

#### Excitement

Throughout the narrated life course, increasing or decreasing Excitement was a relevant theme. Increasing Excitement is related to an expressed feeling of excitation (+): “*Then I went to the brothers, and found everything great at first* “(Daniel, pos. 6) or positive anticipation of what will happen (+): “*We decided to go [to a war and crisis area] we didn’t know where we were going, we just knew we were going [to a war and crisis area]*” (Omar, pos. 58). Another aspect is a general enthusiasm for something (+): “*I wanted to do everything right, do nothing wrong anymore*” (Daniel, pos. 124). Feelings of boredom or disappointed expectations or uncertain view of the future (-) were assessed as expression of decreased Excitement: “*I thought [it] was like that too, you go there, you’re there a few months and then it’s over and you come back, I thought very naively*” (Omar, pos. 200).

#### Anxiety

On the one hand, Anxiety here is understood as a reaction to a genuine risk (+): and on the other hand, as an object indeterminate intrapsychic conflict related to an increasing feeling uncertainty and apprehensions (+): “*I must also say that the fear of being arrested was also already there*” (Omar, pos. 230). This described feeling could also be perceived as decreasing (-): “*but after [some time] you feel, I would say, kind of safe, I didn’t do anything, but still, you think to yourself: OK this thing is over, nothing will happen to me anymore*” (Omar, pos. 232).

#### Pleasure

In distinction to Excitement, a perception of Pleasure is represented by an increasing feeling of joy, desire, or a general striving for Pleasure (+): “*It was cool, relaxed, earphones on, listening to music.*” (Arjan, p. 4). A decreasing feeling was rated when the feeling of Pleasure was absent (-): “*it was just a life situation where I didn’t feel comfortable anymore*” (Daniel, pos. 4) or if a negative feeling was actively perceived (-): “*all the hostility and exclusion that definitely got to me*” (Daniel, pos. 6).

### Case reconstructions

As shown in Figure 1, different intrapsychic phenomena emerged as relevant in the analysis of the three cases. The intrapsychic phenomenon that emerged the most frequently per case was Empowerment (Arjan: 94%; Daniel: 75%; Omar: 75%). For Arjan and Daniel, the next relevant phenomena were Pleasure (Arjan: 89%; Daniel 71%) and Excitement (Arjan: 78%; Daniel; 71%). In contrast, for Omar, Pleasure was only 50% less frequent than Empowerment. His awakening of interest to disengage and his experiences of repression were linked to a decreased feeling of Excitement. For Omar, Excitement was the third frequent phenomenon (55%), whereas the Quest for Significance was in the second place (70%). On the contrary, for Daniel, the Quest for Significance was the less frequent phenomenon (38%) and for Arjan it was in the fourth place (56%). Only for Arjan, the Quest for Belonging (39%) was more frequent than the Sense of Belonging (33%). After his departure to a warzone, Omar perceived just decreasing feelings of a Quest for Belonging. Until then, he had a feeling of being part of a community, which also decreased during disengagement and increased again while (re)building a family. Arjan had an increasing Quest for Belonging since Salafist activism but no strong feeling of actually belonging to a community. Like Omar, he later perceived this while (re)building his own family. For Daniel both, the Sense of Belonging and the Quest for Belonging were relevant throughout his whole pathway. Anxiety was the third frequent phenomenon for Omar (50%), while it was the least frequent for Daniel (38%) and Arjan (28%). For Omar, the perception of Anxiety emerged only after the departure to a war zone. For Daniel, it started after being involved in delinquency for the first time. Arjan is the only one of the cases investigated who experienced Anxiety during the awakening of Islamic interest and re-adaption of Islam.

The reconstruction of the latent structures of meaning and action showed differences in the three cases. Arjan’s intrapsychic phenomena were characterized by an increase in perceptions. Decreasing perception predominantly emerged when the decision making that led to a biographical experience was not made actively. After Daniel’s disruption of Salafist activism and Omar’s awakening of interest to disengage, the intrapsychic phenomena for both were progressively decreasing. For Daniel, similar to Arjan, passive decision making predominantly led to a decrease in Pleasure and Empowerment, as well as an increase in Anxiety and Quest for Significance. As shown in the timeline in Figure 1, Daniel’s Salafist activism lasted the longest, but during that time, he predominantly perceived passive curves of progression. Active decision making was always associated with an increase of Pleasure and often with increasing Excitement. In contrast to Arjan and Daniel, there was no clear connection between passively made decisions and negative feelings in Omar’s case. Passive decision making occurred in Omar’s case only when institutional norms were disrupted and when repression was experienced.

#### Research vignette: The fearless lion

After hearing the opening question of the narrative interview, Arjan offered to report directly from the “real beginning.” He describes a petty criminal youth full of problems and drug consumption. His adolescence is characterized by low impulse control and only poorly developed patterns of action. At the beginning of the opening narration, he had problems giving structure to his narrative. According to the in-depth hermeneutic interpretation group, he talks the freest and did not seem to consciously structure his narration. The interpretation group evaluated this as a sign of “just telling his story without trying to express something.” He triggered the most associations within the interpretation group. From the beginning, he was perceived as “real” and expressing a “bro habitus.” His manifest self-presentation was fearless and independent. In contrast, the analysis of perceived intrapsychic phenomena showed that he certainly was seeking for community. When talking about others, he stages them as necessary to gain something or prove himself. However, a vulnerable aspect becomes evident in his narration of independence. A male member of the interpretation group felt great sympathy for Arjan because “he seems to have a good heart and needs a strong hand and good role models.” However, another female member felt a great antipathy toward him. She felt that femininity was absent from the narrative, as only male imagery was present. However, the way he spoke made it obvious to her that the interviewer was a woman. He often addressed the interviewer directly or included them in the narration and thus made the interviewer part of his enactment of a “bro habitus” in order to prove himself as bold and casual.

As the timeline (Figure 1) shows, Arjan’s awakening of interest in Islam and the awakening of jihadist interests emerged within a year. In the interview, he told how he first found Salafism and how he learned about Islam only later. When this slip of the tongue occurred to him, he broke off the narration at this point and said he would “talk out of order.” Especially in that sequence, it becomes clear that the religious aspect of his activism did not seem to have been a determining factor. When he started to deal with religious issues, increasing Anxiety became a relevant intrapsychic phenomenon. However, Arjan’s behavior, in general, was characterized by a latent desire for Pleasure and Excitement, which he soon found in Salafist activism. He talked about “Jihad, Jihad,” which was perceived as irritating by the interpretation group. For some, it arose an association of a battle cry in the context of a children’s game. Moreover, the entire narration was characterized by a certain lightness despite the serious subject matter. When he bragged about his successes in Salafist activism (e.g., Quran distributions), he spoke of “having been a lion,” which again caused irritation among the interpretation group members. In the Salafist ideology, the lion symbolizes strength and is a frequently used symbol. However, in the interpretation group, this self-description seemed unfamiliar. One group member associated this with comparing how it would be if he had compared himself to another animal “like a deer or a goose,” two animals that she assumed to be very contrary to a powerful lion. She then associated a story she remembered about a tiger that adopted a goose; both got along well. The group was pleased, and then, she added that in the end the goose was accidentally killed by the tiger and ended up being a victim after all. Arjan himself was not talking about joining a terrorist group on a manifest level, as it was something dangerous or serious, an act in which others and one’s own life are endangered. He was talking about it as if it was something that was only possible for him because he was so fearless. However, the association of Arjan being a fearless “goose” that thinks it can live with a “tiger” and tragically ends in death, mirrors some of the neglected seriousness of the subject matter in Arjan’s narration. This defense mechanism was unconsciously adapted by the interpretation group, for example, through irritated laughter at some points.

In the reconstruction of Arjan’s lived experiences, it became clear that Arjan limits his efforts (e.g., by disrupting institutional norms) when he loses Pleasure in something. He is not very ambitious and is focused on the minimum that is required. Sometimes, he has higher goals and a distinct Quest for Significance, which is to live a good life in general. When in doubt, he downgrades his goals without devising an alternative plan of action. In general, he was not successful at the level of institutional norms. Although he sometimes suffered from not feeling empowered, he did not maximize his potential for action, e.g., through further education. In Salafist activism, he already found Pleasure and success, which he attributed to himself and his performance, and therefore felt empowered. By later joining a foreign terrorist organization, he was seeking the opportunity to increase positive feelings of Empowerment and Pleasure independent of institutional norms. Although he manifestly stated helping the *Ummah* (i.e., the Muslim community) and entering paradise as his motivation at that time, these reasons no longer appeared in his reflection, not even for justification reasons.

Not knowing how the interview and Arjan’s story continued, the interpretation group questioned whether Arjan finally left for a war zone. The actual life situation in the war and crisis zone did not correspond to Arjan’s ideas of it. His actual experiences were associated with more displeasure than Pleasure and decreased feelings of expected Excitement. Therefore, in accordance with his already established pattern of action, he decided to break off his stay and return to Germany. Without knowing about the actual process, the group anticipated this development when they wondered about how Arjan’s narration could have ended. They fantasized about big historical events of which people could have been part, but instead decided to have a short look and then did something more pleasurable, while all the other people are at the historical event.

Arjan’s motivation for action, in general, is predominantly self-centered. As for Daniel and Omar, imprisonment was a turning point in his pathway of radicalization. For Arjan, participating in an intervention and exit program was—measured against the limited possibilities in prison—the only perceived option to increase positive feelings and to maintain personal agencies. The motivation to participate in an intervention program can be assessed as intrinsic. However, on a latent level, distancing oneself from ideology was not the original motivating factor to join such a program but to improve his own situation.

After his release from prison, following institutional norms was finally connected to positive feelings. He received extensive support in the subsequent steps of reintegration into society, which he also experienced as helpful.

#### Research vignette: The willing victim

Daniel began his narration by saying he wanted to “go right back” and then argued about his exit process, explaining what made it subjectively impossible for him to leave the Salafist scene. His first sentences triggered feelings of boredom and annoyance in the participants of the interpretation group. He seemed to have started his narration with a clear aim of justification and barely showed any personal agencies. His narration was characterized by passive language (e.g., something happened; one has done something). Instead of feeling sorry for him, the interpretation group felt sorry for the interviewers for being used to stage his victimhood. As in Arjan’s case, the situation was affect loaded, but the atmosphere was more negative. While the discussion about Arjan was rather energetic, in Daniel’s case, the opposite happened, and countertransference of tiredness and depression was perceivable. As shown in Figure 1, Daniel has a strong Quest for Belonging, but also a strong feeling of not belonging to a community that runs throughout his life course. This discrepancy is also reflected in the interpretation group. The interpretation group initially rushed through the case to finish it and talk about the next one. Later, the group mentioned that they felt sorry for Daniel for the way he talks to the interviewers because they could tell he was trying to get a reaction or confirmation from the interviewers that the interviewers were not giving him, at least not on a verbal level. A great feeling of uncertainty and a need for reassurance are evident throughout the interview. In sequences that were not known by the interpretation group, Daniel spoke about the calming effect of reassurance from members of his community. As he recounted his feelings when he felt that he had no support from the community in times of need, it became clear that he had no inherent concept of himself in the outside world. As evidenced by the passivity in his course of life, he was floating until he received stability from the outside. In his childhood, he found much-needed role models outside of his family. He had no inherent feeling of Empowerment. Leaving school and entering work life often represent a change associated with uncertainty. On the one hand, these are important steps in growing up; on the other hand, there is a clear break between the institutional certainty of school and the changes and demands that result from growing independence. During this time, Daniel longed for structures and security that he could not find. Faced with the availability of a religious concept, he converts to Islam. Although his conversion was an active decision, the awakening of interest in Islam was more driven and situational but also connected with an increase in Excitement, Pleasure, Quest for Significance, and Belonging. He later slipped into Salafist activism and was committed for a longer period. He disrupted both social and institutional norms and increased his activism. Daniel longs for institutional structures, but equally wants to stand apart from them. In religious activism, he has found a balance between autonomy and regulated structures. He felt empowered and secured by the Salafist community, in which he found role models like earlier in his childhood. Later, due to decreasing feelings of belonging to the community, he visited other members abroad. This decision was made rather passively, as he did not feel able to solve existing problems by himself and instead had the option of following someone he felt safe with. Regarding his pathway and decision-making processes, he avoids dealing with conflicts and rather changes the “scene” to avoid feelings of displeasure such as emerging boredom due to routine or interpersonal or partnership conflicts. He is striving for new beginnings based on opportunities, driven by external circumstances. He wanted to be part of something without taking responsibility. Even in his retrospection, he was unable to take responsibility for his decisions and behavior. As his decision-making processes reflect, his narration was also very passive. Most aspects of his life just seemed to happen to him. Due to data protection reasons, all references to other persons or names of Salafist groups were anonymized. The interpretation group wondered in what kind of [association] he was active. Without any knowledge of the context, they associated something “provincial” with his narration and imagined how he was active in a “gardening or fishing club.” One member interrupted the group by saying that it is absurd to think about fishing clubs when the topic is terrorism. The associations of the group mirror Daniel’s lack of reflection on his actions. However, without knowing anything about Daniel’s acts or the reason for his conviction, they compared his narration to Adolf Eichmann, a major organizer of the genocide of Jews by the Nazis, and how he was talking about the *Shoa*. The group summarized that Eichmann also had a way of calmly talking about something cruel in such a way that no one would think the subject was that terrible without listening to his exact words. The defense of guilt and shame is inherent throughout the interview. Daniel’s departure to a war and crisis zone followed experiences of repression and was again made rather passively by joining others. His actual engagement in jihadist activism, however, was actively and connected to increasing feelings of Belonging, Empowerment, and Pleasure. The group gives him security. Without the group, a complete collapse of his self-image threatens. He gets into situations that later increase his fears—these are always absorbed by the group, which also legitimizes the respective acts. While being imprisoned after returning to Germany at some point, he struggled with his expectations of the community. In contrast to Arjan and Omar, he was not disillusioned by the actual stay in a war and crisis zone but by disappointed expectations of support by the community while his Quest for Belonging was increasing. He described a process of reflection, but not induced by himself rather than through other people who shared with him their perception of him. Referring to another sequence, the interpretation group concluded that his self-concept seems to be as fragile and fractured as his narration. Besides including himself in a social community, Daniel compensates for insecurities in the context of growing independence and a lack of organizational norms by creating relief through new beginnings including external structures given by this. An external structure that he found in prison was an exit program he attended. Although he later broke with his former community, he nevertheless still latently takes them in protection, which also provided irritating moments in the interpretation group. Referring to the lack of personal agencies in his narration, one group member earlier associated a comparison with the Mafia. Daniel describes himself as if he had simply slipped into something, and that is how it was then. After protecting the group by telling a negative story about some members and then switching to explaining that they made up for it later, she once again picked up the Mafia association and asked herself how certain Daniel could be or feel that he would not be tracked down by “these people” again or maybe he does not completely want to cut the connection for good as he might still feel attached to them.

Not having the feeling of being reintegrated after release from prison, he was integrated into institutional norms that were connected to a feeling of Anxiety and a feeling of not being empowered. However, following social norms was related to positive feelings.

#### Research vignette: The noble spiritual

After the opening question, Omar began to talk by saying “ok, I will start” and then reported on what happened after high school. In contrast to Arjan and Daniel, Omar’s biographical pathway is characterized throughout by a Quest for Significance. It is also characterized by active decisions, and in his narration, he expressed accountability for it. He was striving for a meaningful gain in life and to “do the right thing,” which initially was to have a good education and find a suitable job. Although he had a clear vision of his plans and expected institutional norms, he felt like he did not get the opportunity to implement them. The feeling of Empowerment is relevant throughout Omar’s whole life course. However, Omar was more often feeling not empowered than empowered, which reflects perceived struggles in his life course. Societal marginalization was not manifestly mentioned by him but became apparent in his lived experiences.

He presents himself intellectually and includes the interviewer in his narration. At the beginning of the interview, the atmosphere was more tense than at the end. In the beginning, Omar tries to enact himself as equal to the interviewer. For example, he offers the interviewer to ask him any questions if he should have some and said things like “surely you know about it.” A female member of the interpretation group was wondering whether the interviewer was a man or a woman because she would perceive him as more sympathetic if it had been a man. This expression suggests a perception of “mansplaining” (a description of men explaining things predominantly to women, without being asked), which she would have perceived very negatively. But the interviewer being a man would have had no effect on her sympathies for Omar. Being perceived as intelligent and reflected is important to Omar. The interpretation group experienced him as “pseudo-intellectualizing.” When talking about his increasing interest in Islamic lectures, he mentioned that these were “highly interesting.” The group was irritated about this part, and they imagined how he might have pronounced it. Further on, the interpretation group itself also became part of the enactment when talking about Omar. An interpreter described that Omar presents himself as being spiritual and having a “noble character with which he tries to adorn himself” and has unintentionally adopted his way of speaking.

After Omar’s intellectualization had previously been highlighted as negative, the group itself now discussed the appropriateness of his wording, which was not done in that form in the other cases. A different standard seems to have been applied to Omar’s statements. His self-representation was subject to greater probation pressure than in the other two cases, reflecting a perception that Omar himself seems to have of himself.

While talking about his re-adaption of Islam, he did not directly refer to it as Islam and only said “the Religion” instead. One interpreter mentioned that although he presents himself as well reflected, referring to Islam as “the religion” is not very differentiated. A deep-rooted assumption becomes visible that for Omar, “Islam” is representative of “religion” and that other confessions do not play a role, at least for him. For him, Islam is strongly connected to the Muslim community, with which he identifies himself. He practiced Islam intensively for several years before becoming involved in jihadist issues. Being Muslim represents a high level of morality for him. Being active in Salafist activism like Quran distributions does not seem to have been an important part of his religious practice. He describes it as something he slipped in but not as a fulfillment of a religious duty (i.e., *da’wa*). He also passively justified his awakening of interest to join a jihadist group abroad by saying “only when the Syria conflict came to light in 2012, 2013, (.) my religious/my religion was cold-bloodedly steered in this direction” (Omar, pos. 44). With this, not only has a narrative of innocence been created through an expressed passivity; by saying that his religion has been steered in a certain direction, he makes jihadist commitment a natural part of his religious practice and fully identifies with it. However, engagement in jihadism was also related to increased Excitement and a feeling of Empowerment.

Omar has a strong Sense of Belonging to a community: to his family and the Muslim community. In his narration, the Muslim community and jihadist organizations are conflating. When talking about jihadist organizations, he said that “all Muslims are one.” At this point, it was unclear whether he was distancing himself from the jihadist organization or justified them as part of the Muslim community. In the interpretation group, a discussion was held about how this might be a pattern in religious groups. The discussion led to Catholic church members that were involved in a recent child abuse scandal in Germany. Although this was a very specific topic, the group referred to “the Catholic Church” instead of addressing the offenders, and thus unconsciously adapted the conflating character of Omar’s speech, who consistently spoke of “the religion” and “the Muslims.” Although the two examples initially had little in common, the comparison shows that in both religious groups, individuals have acted criminally but still remain part of their religious community no matter what. This comparison shows that if one fully identifies with the religious community as such, it might be difficult to completely distance oneself from individuals if they are considered part of the community. A strong social component is also reflected in the interview situation, as Omar constantly includes the interviewer in his narration. In the beginning, this was due to a sense of uncertainty. Later, when he learned that he could talk openly and freely with the interviewer, he built up an interplay in which he provoked and included reactions from the interviewer.

However, in addition to a social component, jihadist activism was also something achievable regarding his Quest for Significance. One could assume that it would have fit Omar’s self-presentation of having a “noble character” to join Jihad to provide humanitarian aid. Instead, he was clearly disappointed about missing Excitement and unfulfilled expectations.

From the start of his motivation to join a jihadist group until disengagement, only a short time passed. It was a pleasurable rush into a movement in which he felt belonging and secure, excited, and at the same time feeling like doing the right thing in order to feel personally significant. These perceptions determined his actions and joining a jihadist group and being willing to combat were seen as legitimate ways to satisfy his desires and avoid feeling un-empowered and feeling not excited. His narration was affect loaded, which was perceived and also transferred to the interpretation group. They were wondering about the level of naivety and why Omar assumed that he could have an impact on the situation of the Muslim community when he had not accomplished much else. In fact, he soon got disillusioned in the warzone. Doing combat training increased his feelings of Excitement, Empowerment, and Pleasure, but not being able to implement this, the positive feelings soon decreased. When he could not even find a sense of community and belonging, he soon decided that joining a jihadist group abroad was not the right thing for him. After disengaging from jihadism, Omar’s Quest for Significance nearly vanished and even decreased. Though not manifestly mentioned, a feeling of guilt becomes apparent. After disengaging and returning to Germany, Omar broke up with the community as he no longer felt like he was belonging to it. He completely reintegrated himself into his former social system and completely distanced himself from the Salafist scene and ideology. However, even after disengagement, his general idea of Jihad was still related to Excitement. He wanted to be part of a revolution and still wished it had happened. His regret for joining the Jihad is less intrinsic and more about not being able to make a meaningful change as expected. His increasing anxieties after disengaging were linked to the fear of being arrested or rejected due to his terrorist engagement. He finally found his personal significance in a stable partnership with a woman who was “the right one” and by being able to prove his worth by having a job.

## Discussion and conclusion

As the aim of the present study was to reconstruct the motivational dynamics of engaging in and disengaging from terrorism, we presented a multi-methodical procedure based on three narrative interviews of German former Salafist jihadists, combining a biographical reconstruction of the lived experiences with a psychoanalytically informed interpretation of the lived experiences.

It could be shown that all three cases were driven differently by perceived intrapsychic phenomena and have shown different personal agencies. General connections between those intrapsychic phenomena and personal agencies could be drawn. However, it became clear that their individual pathways were conflictual. In Figure 1, those conflicts are illustrated. In each case contradictory desires and perceptions of cognitive elements were present. However, the biographical pathways of (de)radicalization showed different dynamics of increasing or decreasing perceptions of intrapsychic phenomena over time. Perceiving a discrepancy of these cognitive elements is called cognitive dissonance, a conflict individuals strive to bring back into balance (Festinger, 1962; Maikovich, 2005). This is, for example, observable in Figure 1 when the direction of the personal agencies (active vs. passive decision making) changes regarding the described biographic experiences, in which the interview candidates expressed to have attempted to change patterns of action self-initiated. Furthermore, in all three cases cognitive dissonances were observable regarding their perceived intrapsychic phenomena, but not in the same areas. It became visible that they were driven by different needs and that they were characterized by different dynamics. Nilsson (2022) argues that cognitive dissonance is especially related to goal-driven behavior and concludes that perceived cognitive dissonance is a causal mechanism “that can explain how individuals with collective identities can be motivated to opt for jihad” and how motivations change (p. 14). This case study suggests that there is not one focal goal or motivation that drives individuals to engage in and disengage from terrorism, but a combination of multiple aspects that result from the individual’s life course. Therefore, we also assume that concepts such as cognitive dissonance should be considered in a more differentiated way and rather as a representation of mental states than as a causal mechanism. However, the self-presentation of the three examined cases allows conclusions to be drawn about the self-chosen retrospective motives for their commitment to a terrorist group, which we differentiated from their overall motivational dynamics. To roughly summarize the core aspects: Arjan, who presented himself as fearless and powerful on a manifest level, was striving for a good life and belonging to something on a latent level. Daniel was placing himself as a victim of external circumstances. He was seeking to be part of a community and felt anxious without their safety-giving environment. Omar, creating a spiritual and noble image of himself, wanted to be someone and do something significant, while feeling suppressed by external circumstances.

### Discussion of intrapsychic phenomena

It is necessary to understand what drives a person’s decision making and behavior, and it might help to understand why different individual factors cumulate to the same result of joining terrorist groups in some cases, while the same factors do not in many more. Applying psychoanalytically induced interpretation in an in-depth hermeneutic setting, we were able to demonstrate that the biographical pathways of the three cases studied were diverse and that different perceptions of feelings, desires, and needs were differently relevant to them.

Based on reflections on their field work, Lloyd and Kleinot (2017) identified three terrorist pathway types that are roughly comparable with the results of the present multiple case study. They identified terrorists as having a “noble cause,” as “criminals” or “pathological narcists.” However, the present case study comes to different conclusions and empirically analyzed the intrapsychic dynamics of terrorists throughout their entire biographical pathways. While “criminal” or “pathological narcissism” characterizes a personality type, “noble cause” characterizes a motive to engage in terrorism. Based on this case study, we assume that this is primarily limited to one’s self-representation at a given point in time and that the totality of motivational factors over a person’s life course must be included to gain a comprehensive understanding of a person’s motivation to engage or disengage in and from terrorism.

In the analyzed cases, the concept of being part of a group had different meanings for the individuals. For one, the group was a necessary reference frame for self-exaltation; for another, it was a safety-giving structure, while the third person was moreover identified with it. Fonagy (cited in Hough, 2004, p. 824) indicates that being a member of a large group activates the attachment system, which affects biological processes that provide a feeling of belonging and safety. This case study showed that Quest for Belonging and actual Sense of Belonging were relevant to all three cases (see also Hofinger and Schmidinger, 2020). In two of the cases, the group in which they practiced Salafist activism in Germany was different from the group they ended up in the war and crisis zone. For them, it was easier to leave the terrorist group abroad and focus on personal needs. The other case just followed members of his group and thus never lost his Sense of Belonging and safety until losing connection to the group due to police intervention. When separated from their initial group and their reference persons, it was easier for them to distance themselves from both the jihadist group and ideology. Referring to Fonagy (cited in Hough, 2004), that might indicate that at least two cases were attached to their Salafist group, but not to the overall Muslim community (i.e., the Ummah), as they presented it. This seems to be a major difference between those terrorists who disengaged and those who remained in the terrorist organization. The Quest for Belonging and the actual Sense of Belonging to a group or community were perceived differently. While one case had a stronger Quest for than a Sense of Belonging, another had more frequent feelings of belonging than the quest for it, while the third case had nearly no relevant Quest for Belonging because he felt sure about his belonging to the Muslim community until his awakening of interest to disengage.

According to the model of radicalization and deradicalization by Kruglanski et al. (2014, p. 80), the quest for personal significance is the motivational element toward violent extremism and the beginning of the radicalization process. Compared with Kruglanski et al. (2014), our concept of the Quest for Significance is more detailed and not related to a focal goal but related to specific experiences that were also relevant long before the radicalization process. However, the Quest for Significance does play a relevant role in the three analyzed cases throughout their life courses. Kruglanski et al. (2014) included Empowerment as part of the personal quest for significance. In our case study, Empowerment emerged as an own main theme, which occurred even more frequently than the Quest for Significance.

Particularly, the phase from childhood to adolescence is characterized by transitions. Hence, old ideals are disappearing and the need for new ideals arises in this phase, Benslama (2017, p. 41) describes this with reference to Winnicott as de-idealization. This perceived state of degradation of the self and feelings of emptiness and depression is transformed in the phase of re-idealization, which is associated with new developed ideals, exaggeration of one’s own self-representation and the feeling of a passionate awakening (Benslama, 2017, p. 42). In the present case study, this description applies to both the process of awakening of interest in Islam followed by Salafist and jihadist activism (re-idealization) and the process of disengagement from the group and ideology (de-idealization), followed by the desire to live a social and norm conform life in society (re-idealization). Benslama (2017, p. 36) further pictures the (re)adaption of religion as an uprising lift of narcissism, which is relatable to our concept of feeling empowered. Additionally, the image of a lift well symbolizes the rapid fall of Excitement and decreasing feelings of Empowerment when experiences did not correspond to what the individuals expected. Moreover, the fact that all three returned after only a short stay in the war and crisis zones is a sign of ambiguity intolerance. They did not find what they expected and came back. With a higher tolerance to ambiguity, they might have adapted to the situation and found new ways of signification. However, they returned. The aspect of ambiguity intolerance should also be taken into account as a relevant psychodynamic aspect in intervention and reintegration measures, especially for returnees from warzones.

The feeling of Empowerment was widely associated with Excitement. In an experimental study, Schumpe et al. (2018) showed that the psychological mechanism of sensation seeking is a predictor of support for violent activist groups. Although we have not estimated a general tendency to sensation seeking but have analyzed perceived feelings of increasing or decreasing Excitement for experiences over the life course, our study supports the results presented by Schumpe et al. (2018). The (re)adaption of Islam and integration into the Salafist community, as well as joining terrorist groups, was in all cases related to increasing Excitement. Disappointed expectations and decreasing perceptions of Excitement were initial drivers to decide to disengage from jihadist activism. This was true for leaving the terrorist organization abroad and at home, as well as for disengaging from jihadist ideology in general. Excitement also played a role in attending exit programs in prison.

Anxiety is a factor that is seldom considered in radicalization dynamics. Related to the three phases of the staircase model of terrorism (Moghaddam, 2005), Ljamai (2020) identified three different kinds of fear relevant to the radicalization process, namely, fear of victimization, fear caused by guilt feelings, and fear of being controlled by hatred and revenge. In this case study, we went beyond the pathways of radicalization and included the time before Salafist activism, as well as after disengagement in our analysis. Our results suggest that the feeling of Anxiety should be conceptually separated from the radicalization process, as Anxiety is a dynamic concept. In the analyzed cases, changes in increasing and decreasing perceived Anxiety were visible. However, related to internal or external circumstances, changes also occurred within a single phase and were related to explicit feelings of Anxiety, such as fear of repression or rejection, or more abstract feelings, such as worries about the future. In this case study, victimization rather was a concept that was provoked by the individuals, such as deliberately provocative behavior that resulted in repression, or it was manifestly expressed to justify one’s behavior or actions. In one case, Anxiety was relevant while adapting Islam, but in all cases, Anxiety increased after engaging in jihadist activism, which was mainly due to personal uncertainty regarding the risk of criminal prosecution.

In psychoanalytic theory, Pleasure is a complex experience and an inherent core driver of behavior. A basic objective of humans is to obtain Pleasure and avoid displeasure (Freud, 1920). In our analysis, we conceptualized Pleasure as less theoretical and more focused on the concrete perception of it. In all cases, a frequent link was observed between the decrease in Pleasure and passive decision making. Furthermore, the decrease in Pleasure was frequently associated with an increasing Quest for Belonging and perception of Anxiety, as well as decreased perceptions of Excitement and Empowerment. These results fit well with the assumption of Moccia et al. (2018, p. 359) who describe Pleasure as “beyond the pleasure principle” of Freud, as independent of libido, which includes, for example, a linkage with objects such as “perception of self-efficacy, cultural significance, and the propensity to communicate and form interpersonal relationships.” An expressed passive decision-making process is accompanied by a low perception of self-efficacy and personal agencies. Referring to Freud (1924), Moccia et al. (2018) conclude that Pleasure is connected to pain and thus carries a potential destructive component. In other words, individual radicalization processes may embody a potential self-destructive tendency that roots in Pleasure and results in pain (Benslama, 2017, p. 37).

### Discussion of implications

The needs addressed in the exit programs are diverse. However, from this study with a comparatively small sample, it can be deduced that it is helpful to provide different concepts for exit programs, as is the case in Germany. In some cases, it is helpful when the exit consultants address the individual directly, whereas in other cases, it is more helpful if the individual intrinsically chooses to contact such a program. However, even if the motive is to increase feelings of Excitement due to monotonous everyday life in prison but not due to an initial ambition to distance oneself from extremism, these programs can still have a peripheral effect on processes of disengagement and distancing from terrorist groups and ideology. In the presented cases, the exit programs helped especially with finding back to institutional norms, which seems to be a core factor for reintegration into society. All three cases benefited from a social system they could rely on after release from prison. Compared with a case study with a wider sample by Emser et al. (2022), the benefit of having a non-radical social system is a relevant factor in the process of distancing and reintegration. However, due to not being able to interview former extremists without a non-radical social system, we cannot draw conclusions about whether the absence of such hinders the process. However, Emser et al. (2022) could show that staying in a radical social system indeed is an impediment to distancing and reintegration, which is also true for the present case study. Furthermore, this case study has shown that motivational dynamics are emerging throughout the lifetime of individuals on both a conscious and unconscious level. Along their life path, different needs or feelings were more important or receded into the background at different stages of life, but it could be shown that they did not vanish. In other words, the case characteristics and motivational dynamics are independent of the (de)radicalization process itself, although they both influence it and get influenced by it. We argue that a psychotherapeutic and moreover a psychodynamic approach should be included in exit programs. Helping people to reintegrate into society, work–life, and their families are significant factors. However, stabilizing the ego- and psychosocial functions also seems rewarding for successful reintegration. In some German federal states, but not at a nationwide level, such an approach is applied in the form of special trainings in the field of radicalization prevention and distancing programs. Access is assigned, for example, directly in the penal system, but also *via* judicial directives (see, e.g., Krause and Friedmann, 2021). Another approach specifically offers psychotherapy as a contribution to reintegration (Violence Prevention Network, 2020). In this case study, we could show based on only three cases how different individuals cope with their radical past and how their actual needs differ even after disengaging and the willingness to distance themselves from the terrorist group and ideology. Even if trauma was not an issue in the cases we investigated, traumatic experiences will probably play an increasing role, especially for future returnees. We could also show that also phenomena such as shame, guilt, depressiveness, or attachment are topics worth addressing in the reintegrating process. We consider it advisable to implement such psychodynamic approaches nationwide and outside the penal system.

### Limitations

In biographical research, there is a discourse on the significance of narrated life stories. Authors such as Frisch (1968) or Osterland (1983) consider the narrated life story as a kind of subjectively created illusion. Schütze (1981) and Rosenthal (1995, p. 17) pursue the approach that the biographical past manifests itself in the presence of the narrative. The narrated and experienced life history thus form a mutually constituting relationship. Through triangulation of data and methods, we attempted to objectify the experienced life. However, no general truth is aimed at, but rather an attempt was made to examine perspectives of the constituting relationship between experienced and narrated life history to understand radicalization and deradicalization in the holistic context of a person’s life course and perception. This qualitative case study was based on a multi-methodological approach that combined different research methods and questions to provide holistic case reconstructions at different levels of interpretation. Methodologically and theoretically, this study was psychoanalytically framed to examine both the conscious and unconscious levels of experiences and dynamics. Therefore, the objective was not to test theories but to explore lived experiences and own contexts of interpretations of former jihadists to expand knowledge about relevant dynamics in the field of radicalization and distancing from terrorist groups and ideology. The selected cases and the sample size were sufficiently large to introduce this complex approach and identify relevant aspects of motivational dynamics for three paradigmatic cases. We do not claim completeness of the intrapsychic phenomena that we have identified as relevant for these cases. Other phenomena may emerge as relevant in a wider sample. For example, it is reasonable to assume that the dynamics are different for women who made up at least 25% of German citizens who departed to a war zone to join a terrorist group abroad (Bundesamt für Verfassungsschutz, 2022). Hence, it would be beneficial to apply the presented approach to a broader sample size and include women. To provide a contrast, cases could also be included who have not returned and are currently still in the war and crisis zone (see, e.g., Hofinger and Schmidinger, 2020). Further research on the identified intrapsychic phenomena should be conducted to examine the relevance in more detail. Concluding, we see great potential in psychodynamically and psychoanalytically induced approaches to understanding processes and pathways of (de)radicalization. The added value of such an approach lies not only in the in-depth analysis of a case and decoding of the latent meaning, but also in the specific reflection of the interpretation process as such. The reflection of the subjectivity of the researchers thus goes beyond the value of a quality criterion of qualitative research and additionally becomes an instrument of insights (in German *Erkenntnisinstrument*).

## Data availability statement

Due to data protection and the high sensitivity of the topic, the data cannot be made available. Requests to access these datasets should be directed to MM, mika.moeller@fu-berlin.de.

## Ethics statement

The research project “PrADera” from which the data in this manuscript originated was based at Technische Universität Berlin. The studies involving human participants were reviewed and approved by the German Federal Agency for Migration and Refugees. The participants provided their written informed consent to participate in this study. In the scope of the doctoral project of the first author, this study complies with the requirements of the institutional data protection office of the Technische Universität Berlin and the GDPR, respectively.

## Author contributions

MM: conceptualization, methodology, investigation, writing – original draft preparation, and visualization. PL: validation and writing – reviewing and editing. HS: writing – reviewing and editing. All authors contributed to the article and approved the submitted version.
